# The GH130 Family of Mannoside Phosphorylases Contains Glycoside Hydrolases That Target β-1,2-Mannosidic Linkages in *Candida* Mannan*

**DOI:** 10.1074/jbc.M115.681460

**Published:** 2015-08-18

**Authors:** Fiona Cuskin, Arnaud Baslé, Simon Ladevèze, Alison M. Day, Harry J. Gilbert, Gideon J. Davies, Gabrielle Potocki-Véronèse, Elisabeth C. Lowe

**Affiliations:** From the ‡ Institute for Cell and Molecular Biosciences, Medical School Newcastle University, Newcastle upon Tyne NE2 4HH, United Kingdom,; § Université de Toulouse, INSA/UPS/INP, LISBP, F-31077 Toulouse, France,; ¶ CNRS, UMR5504 and; ‖ INRA, UMR792 Ingénierie des Systèmes Biologiques et des Procédés, F-31400 Toulouse, France, and; the ** York Structural Biology Laboratory, Department of Chemistry, University of York, York YO10 5DD, United Kingdom

**Keywords:** enzyme, glycosidase, glycoside hydrolase, microbiome, phosphorylase, x-ray crystallography

## Abstract

The depolymerization of complex glycans is an important biological process that is of considerable interest to environmentally relevant industries. β-Mannose is a major component of plant structural polysaccharides and eukaryotic *N*-glycans. These linkages are primarily cleaved by glycoside hydrolases, although recently, a family of glycoside phosphorylases, GH130, have also been shown to target β-1,2- and β-1,4-mannosidic linkages. In these phosphorylases, bond cleavage was mediated by a single displacement reaction in which phosphate functions as the catalytic nucleophile. A cohort of GH130 enzymes, however, lack the conserved basic residues that bind the phosphate nucleophile, and it was proposed that these enzymes function as glycoside hydrolases. Here we show that two *Bacteroides* enzymes, BT3780 and BACOVA_03624, which lack the phosphate binding residues, are indeed β-mannosidases that hydrolyze β-1,2-mannosidic linkages through an inverting mechanism. Because the genes encoding these enzymes are located in genetic loci that orchestrate the depolymerization of yeast α-mannans, it is likely that the two enzymes target the β-1,2-mannose residues that cap the glycan produced by *Candida albicans*. The crystal structure of BT3780 in complex with mannose bound in the −1 and +1 subsites showed that a pair of glutamates, Glu^227^ and Glu^268^, hydrogen bond to O_1_ of α-mannose, and either of these residues may function as the catalytic base. The candidate catalytic acid and the other residues that interact with the active site mannose are conserved in both GH130 mannoside phosphorylases and β-1,2-mannosidases. Functional phylogeny identified a conserved lysine, Lys^199^ in BT3780, as a key specificity determinant for β-1,2-mannosidic linkages.

## Teaser

**Background:** A cohort of a family of mannose phosphorylases lack phosphate binding residues, suggesting that they display non-phosphorylase activities.

**Results:** The non-phosphorylase enzymes were shown to be β-mannosidases.

**Conclusion:** Replacing basic phosphate binding residues with carboxylic amino acids converts mannoside phosphorylases into glycoside hydrolases.

**Significance:** Functional phylogeny can be used to distinguish between closely related glycan phosphorylases and glycoside hydrolases.

## Introduction

The microbial recycling of complex glycans is an important biological process that plays a central role in the carbon cycle. The process is also of significant industrial interest particularly in the biofuel and biorefinery sectors (1). The cleavage of the glycosidic bonds in complex glycans is primarily mediated by glycoside hydrolases, although polysaccharide lyases and, to a lesser extent, glycan phosphorylases contribute to the degradative process (2). These carbohydrate active enzymes or CAZymes are grouped into sequence-based families in the CAZy database (3). Although enzymes in the same family may display different substrate specificities, the fold, catalytic mechanism, and catalytic apparatus is conserved in the vast majority of the 133 glycoside hydrolase (GH)^3^ families (3).

**FIGURE 1.**
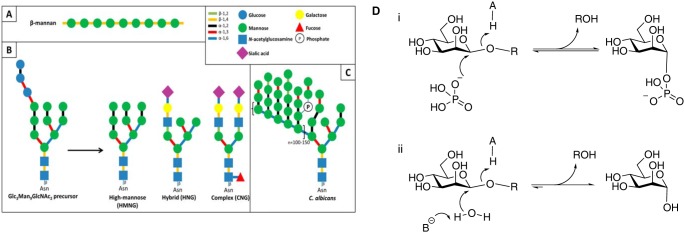
**Schematic representation of the mannosides present in the human gut and reaction mechanisms of inverting glycoside phosphorylases and hydrolases.** *A*, plant β-mannan, a hemicellulosic component of plant cell walls. *B*, human *N*-glycans, showing the different types of structures found on mature glycoproteins formed from a common Glc_3_Man_9_GlcNAc_2_ precursor. *C*, yeast mannan. The structure depicted here is that of *C. albicans*, containing β-1,2-linked mannosyl units that cap the side chains of the α-1,6-mannose backbone. *D*, general reaction mechanisms for inverting mannoside phosphorylases (*panel i*) and hydrolases (*panel ii*).

Mannose-containing glycans are important components of the secondary cell walls of many plants. These plant mannans are homopolymers of β-1,4-Man units that can be decorated at O_6_ with α-galactose residues and are thus further defined as galactomannans (Fig. 1*A*). In glucomannans, the backbone consists of random sequences of β-1,4-linked Man and Glc residues (4). The core of mammalian *N*-glycans contains a conserved Man-β1,4-GlcNAc linkage, whereas α-linked Man units are commonly found elsewhere in these structures (5) (Fig. 1*B*). The proteins in yeast cell walls contain particularly complex *N*-glycans referred to as α-mannans in which the core *N*-glycan is extended by ∼200 α-1,6-Man units that are also decorated with α-linked Man side chains (6). In some yeasts, such as the human pathogen *Candida albicans*, β-1,2-mannose residues cap the α-mannans side chains, and extended β-1,2-Man chains are attached to the α-mannan via phosphate bridges (Fig. 1*C*). β-1,2-Man units that play an important role in detection of *C. albicans* by the innate immune system via the C-type lectin, galectin-3 (7, 8). β-1,2-Mannans also fulfill an important storage role in *Leishmania* parasites (9). Plant cell wall mannans are hydrolyzed by GH5, GH26, and GH113 β-mannanases, whereas β-1,4-mannosides are targeted primarily by enzymes in GH2 and GH5 (see Ref. 2 for review). These enzymes display a (β/α)_8_ fold and cleave glycosidic linkages by a double displacement mechanism leading to retention of anomeric configuration (10–12).

GH130 contains exo-acting β-mannoside phosphorylases that cleave the β-1,2- and β-1,4-mannosyl linkages between Man and Man, Glc, or GlcNAc residues (13–18). The phosphate nucleophile attacks C1 of the Man in the active site below the α-face of the pyranose ring leading to phosphorolysis. The single displacement mechanism displayed by these phosphorylases leads to inversion of anomeric configuration (16). The phosphate is positioned in the active site through polar interactions with three basic residues that are highly conserved within GH130 (19, 20). A subset of GH130 members, however, lacks these basic residues, and it was proposed that these enzymes may function as glycoside hydrolases cleaving mannosidic bonds through a hydrolytic reaction (18).

To test the GH130 glycoside hydrolase hypothesis, the structure and biochemical properties of a representative GH130 enzyme were determined. The enzyme, BT3780, which lacks the canonical phosphate-binding basic residues, is up-regulated by the dominant human gut bacterium *Bacteroides thetaiotaomicron* (21) in response to yeast mannan. BT3780 is encoded by a polysaccharide utilization locus (PUL) inthe *B. thetaiotaomicron* genome that orchestrates the depolymerization of yeast mannan. A similar PUL encodes BACOVA_03624, one of seven GH130 proteins of the human gut symbiont *Bacteroides ovatus* ATCC 8483. The crystal structure of BACOVA_03624 was released in the Protein Data Bank (PDB) in 2011 (code 3QC2), but to date no function had been attributed to this protein. Thus, a potential substrate for these enzymes is the Man-β1,4-GlcNAc linkage at the base of *N*-glycans or the β-1,2-Man units that cap the side chain of some fungal α-mannans. The data, presented here, showed that BT3780 and BACOVA_03624 are exo-acting glycoside hydrolases that cleave Man-β1,2-Man linkages using water, not phosphate, as the nucleophile in a single displacement reaction. The three-dimensional structure of the enzymes in concert with function phylogeny identified motifs within GH130 that delineate glycoside hydrolases and enzymes, both hydrolases and phosphorylases, that target β-1,2-mannosidic linkages.

## Materials and Methods

### 

#### 

##### Cloning, Expression, and Purification of Bacteroides GH130 Enzymes

BT3780 was amplified from *B. thetaiotaomicron* genomic DNA and cloned into pET28a with an N-terminal His_6_ tag using NheI and XhoI restriction sites. To generate DNA encoding, the sequence of the protein was used as template for gene synthesis with codon optimization for *Escherichia coli* heterologous production (Biomatik, Cambridge, Canada) and was subsequently cloned into the pET28a vector. The two genes were expressed in *E. coli* BL21 cells transformed with the appropriate recombinant plasmids. The recombinant *E. coli* strains were cultured in Luria broth supplemented with 50 μg/ml kanamycin. Cultures cells were grown at 37 °C to mid-log phase and induced with 1 mm isopropyl β-d-1-thiogalactopyranoside at 16 °C overnight. Cells were harvested by centrifugation 5000 rpm for 5 min and resuspended in 20 mm Tris-HCl buffer, pH 8.0, containing 300 mm NaCl (buffer A). Cells were lysed by sonication, and the cell-free extract was recovered by centrifugation at 13,000 rpm for 30 min. BT3780 was purified from the cell-free extract using immobilized metal affinity chromatography using Talon^TM^, a cobalt-based matrix. Proteins were eluted from the column in buffer A containing 100 mm imidazole. For crystallization trials, immobilized metal affinity chromatography-purified protein was concentrated and further purified by gel filtration chromatography using a Superdex S200 16/600 column equilibrated in Buffer A.

##### Purification of Mannan from C. albicans and β-1,2-Manno-oligosaccharide Production

*C. albicans* strain JC747 (SN148 (*arg4*Δ/*arg4*Δ *leu2*Δ/*leu2*Δ *his1*Δ/*his1*Δ *ura3*Δ::*imm^434^*/*ura3*Δ::*imm^434^ iro1*Δ::*imm^434^*/*iro1*Δ::*imm^434^*) CIp30 (*URA3 HIS1 ARG4*) was grown in YPD medium (22) at 30 °C. Mannan was purified from *C. albicans* cells as follows. Cell pellets were resuspended in MilliQ water and autoclaved at 121 °C for 3 h. Mannan was precipitated with 4 volumes of ice cold ethanol. Precipitate was pelleted by centrifugation at 5000 rpm for 10 min, resuspended in water, dialyzed overnight against MilliQ water, and freeze dried to remove residual ethanol. To produce β-1,2-manno-oligosaccharides, *Candida* mannan at 5 mg/ml was acid-hydrolyzed with 10 mm HCl at 100 °C for 1 h. The acid hydrolysis was neutralized with sodium hydroxide, oligosaccharides were purified by size exclusion chromatography using P2 Bio-Gel P2 (Bio-Rad) columns, and the oligosaccharides were eluted in MilliQ water.

##### Enzyme Assays

All enzyme assays unless otherwise stated were carried out in 20 mm Na-Hepes buffer, pH 7.5., containing 100 mm NaCl. Assays were carried out with 1 μm BT3780 against 1 mg/ml substrate at 37 °C for up to 16 h. Aliquots were taken over a 16-h time course, and samples and products were assessed by TLC and high pressure anion exchange chromatography (HPAEC) with pulsed amperometric detection. Sugars were separated on a Carbopac PA200 guard and analytical column in an isocratic program of 100 mm sodium hydroxide. Sugars were detected using the carbohydrate standard quad waveform for electrochemical detection at a gold working electrode with an Ag/AgCl pH reference electrode. Kinetic parameters were determined using the d-mannose detection kit from Megazyme International, measuring the release of mannose at absorbance of 340 nm. To determine kinetic parameters, 2 μm of BT3780 was assayed against varying concentrations of polysaccharide or oligosaccharides between 0.1 and 2 mm. Mannose release was measured, and the values were plotted using linear regression giving *k*_cat_/*K_m_* as the slope of the line. Mutants were assessed for activity against *C. albicans* mannan at 1 mg/ml with varying enzyme concentrations between 1 and 200 μm. All assays were carried out in triplicate.

##### NMR Spectroscopy

Reaction buffer 10× (1×, 20 mm sodium phosphate buffer, pH 7.5, containing 100 mm NaCl) and *C. albicans* mannan were freeze dried and resuspended in D_2_O twice prior to the experiment. BT3870 was transferred to reaction buffer in D_2_O by extensive buffer exchange. Initial spectra were recorded of 900 μl of 10 mg ml^−1^ *C. albicans* mannan in reaction buffer before initiating the reaction by the addition of 100 μl of BT3780 (final concentration, 30 μm). ^1^H NMR spectra were recorded in D_2_O on a Bruker Avance III HD 500 MHz NMR spectrometer operating at 500.15 MHz at regular intervals. The chemical shift is quoted in ppm relative to tetramethylsilane, and each spectrum was acquired with 16 scans. Spectra of mannose and mannose-1-phosphate (2.5 mm in reaction buffer) standards were also recorded.

##### Crystallography

BT3780 purified by immobilized metal affinity chromatography and size exclusion chromatography was concentrated in a 30-kDa cutoff centrifugal concentrator and buffer-exchanged into H_2_O prior to crystallization. Crystals were obtained in 2.2 m NH_4_SO_4_, with 0.3 m mannose in 96-well sitting drop TTP Labtech plates (200-nl drops) with BT3780, at 15 mg/ml. Crystals were cryoprotected with saturated NH_4_SO_4_, and data were collected at Diamond Light Source on Beamline I04-1 (λ 0.92 Å) at 100 K. The data were integrated with XDS (23) and scaled with Aimless (24). Five percent of observations were randomly selected for the *R*_free_ set. Space groups were determined using Pointless (25). The phase problem was solved by molecular replacement using the program Molrep (47) and the PDB search model 3QC2. Initial phases were used in the program Buccaneer (25) to automatically build the model. Solvent molecules were added using COOT (26) and checked manually. The model underwent cycles of model building in COOT (26) and refinement in Refmac (27). All other computing used the CCP4 suite of programs (28).The model was validated using MolProbity (29), and data statistics and refinement details are reported in Table 1. The model was deposited in the PDB and given the code 5A7V. Structure representations were made in PyMOL (version 1.7.4, Schrödinger).

**TABLE 1:** **Data statistics and refinement details** The values in parentheses are for the highest resolution shell.

	BT_3780
**Data statistics**	
Beamline	IO4-1
Date	22/05/14
Wavelength (Å)	0.92
Resolution (Å)	46.58–1.35 (1.37–1.35)
Space group	P2_1_22_1_
Unit-cell parameters	
*a* (Å)	75.77
*b* (Å)	118.1
*c* (Å)	126.6
α-β-γ (°)	90–90–90
Unit cell volume (Å^3^)	11,131,912
Solvent content (%)	63
No. of measured reflections	827,701 (40,692)
No. of independent reflections	243,880 (12,061)
Completeness (%)	98.3 (98.8)
Redundancy	3.4 (3.4)
*R*_merge_ (%)	4.9 (52.8)
<I>/<σ(I)>	10.7 (1.8)

**Refinement statistics**	
*R*_work_ (%)	13
*R*_free_ (%)*^a^*	15
No. of non-H atoms	
No. of protein, atoms	5908
No. of solvent atoms	907
No. of ligand atoms	72
Root mean square deviation deviation from ideal values	
Bond angle (°)	0.014
Bond length (Å)	1.7
Average B factor (Å^2^)	
Protein	15.9
Solvent	35
Ligand	14
Ramachandran plot, residues in*^b^*	
Most favored regions (%)	100

*^a^* 5% of the randomly selected reflections excluded from refinement.

*^b^* Calculated using MolProbity.

## Results

### 

#### 

##### Biochemical Properties of BT3780

**FIGURE 2.**
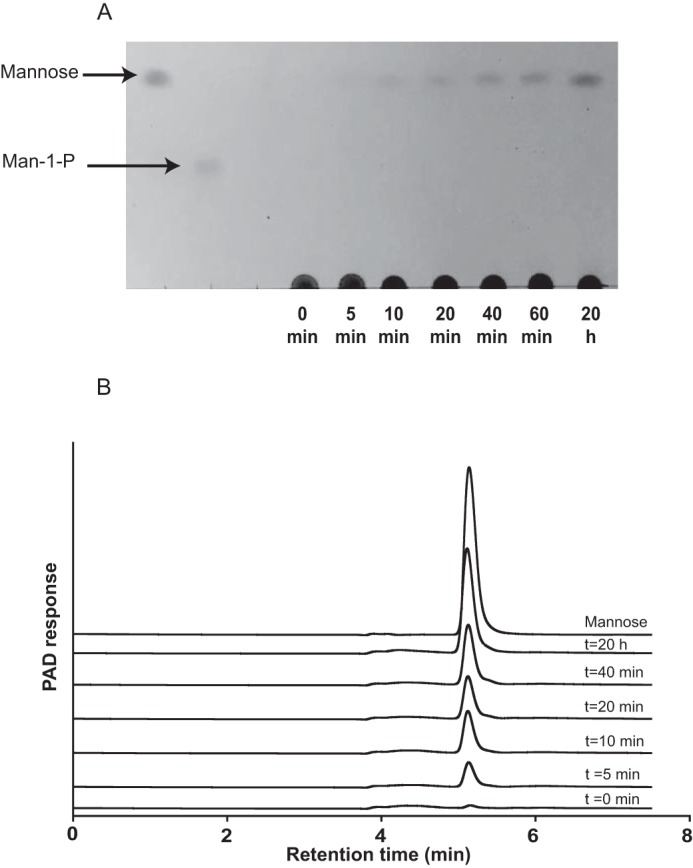
**TLC and HPAEC-PAD analysis of BT3780 catalyzed reactions.** BT3780 was incubated with 1 mg/ml *C. albicans* mannan for up to 16 h as described under “Materials and Methods.” Samples were removed at regular intervals and analyzed by TLC (*panel A*) and HPAEC (*panel B*). *PAD*, pulsed amperometric detection.

**FIGURE 3.**
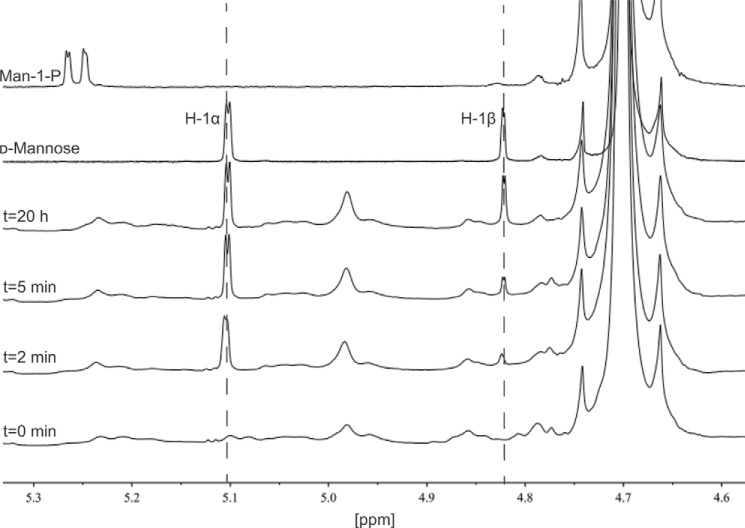
**NMR analysis of the activity of BT3780.** ^1^H NMR spectra were recorded in D_2_O on a Bruker Avance III HD 500 MHz NMR spectrometer operating at 500.15 MHz. The chemical shift is quoted in ppm relative to tetramethylsilane, and each spectrum was acquired with 16 scans. The spectrum of 10 mg/ml *C. albicans* mannan in 20 mm sodium phosphate, pH 7.5, 100 mm NaCl, was recorded prior to and after addition of 30 μm BT3780, at the time points indicated on the graph. A peak at the chemical shift corresponding to the H-1α proton of mannose (5.10 ppm) was detected after 2 min, and mutarotation to the β-anomer was observed subsequently (4.82 ppm), indicating that the reaction proceeds with inversion of anomeric configuration. Spectra of mannose and mannose-1-phosphate (anomeric hydrogen 5.28 ppm) in reaction buffer are also shown.

The genome of *B.thetaiotaomicron* contains three PULs: MAN-PUL1, MAN-PUL2, and MAN-PUL3, which orchestrate the degradation of yeast α-mannan (21). Within MAN-PUL2 is a gene encoding BT3780, a member of CAZy family GH130. This family currently comprises β-d-mannoside phosphorylases that mediate bond cleavage through a single displacement mechanism with phosphate as the nucleophile, resulting in the inversion of anomeric configuration and the generation of α-mannose-1-phosphate. Thus, the possible linkages in *Saccharomyces cerevisiae* α-mannan targeted by BT3780 are Man-β1,4-GlcNAc (possibly Man-β1,4-GlcNAc-β1,4-GlcNAc) and α-Man-1-phosphate (where cleavage would then be associated with a reverse phosphorolysis reaction). The data presented in Table 2 show that BT3780 displayed no *exo*- or *endo*-activity against these linkages in the presence or absence of phosphate. Yeast and fungal α-mannans are not all identical, and in the *C. albicans* α-mannan the mannose side chains are capped by one or more β-1,2-mannose units, which are therefore also potential substrates for BT3780. Incubation of *C. albicans* α-mannan with BT3780 released a product that co-migrates with mannose on HPAEC and TLC (Fig. 2), suggesting that the enzyme indeed targets these β1,2-mannose units. Furthermore, the inability of BT3780 to release mannose from *S. cerevisiae* mannan, which lacks the capping β-Man residues, is also consistent with this proposed specificity for β1,2-mannosyl linkages. The specificity of BT3780 for β-1,2-Man linkages was further supported by the observation that 212 and 620 μmol of mannose were released from 0.5 mg of *C. albicans* α-mannan by the side chain cleaving α-mannosidase, BT3774 (21), before and after treatment of the glycan with BT3780, respectively. Several additional lines of evidence showed that BT3780 generated mannose and not mannose-1-phosphate. Thus, the activity of BT3780 could be monitored by standard mannose detection kits in which mannose-1-phosphate is not a substrate for the linker enzymes (Megazyme International d-mannose/d-fructose/d-glucose assay kit); NMR spectra of the sugar released by BT3780 revealed H^1^ signals corresponding to both the α and β anomers of mannose, whereas the signal for the anomeric hydrogen in mannose-1-phosphate, with a chemical shift of 5.28 ppm, was absent (Fig. 3). Thus, the enzyme does not mediate phosphorolysis or reverse phosphorolysis reactions and therefore is not a glycoside phosphorylase. These data instead show that BT3780 is an exo-acting glycoside hydrolase that hydrolyzes β1,2-mannosidic linkages. The observation that the enzyme displayed no detectable activity against mannose-linked β1,4-linked to mannose or glucose (in mannans and glucomannans, respectively) or GlcNAc (Table 2) confirms its tight specificity for β-1,2-mannosidic linkages.

**TABLE 2:** **Catalytic activity of GH130 β-mannosidases**

Enzyme	Substrate	Activity (*k*_cat_/*K_m_*)
		*min*^−*1*^ *m*^−*1*^
BT3780	*Candida* mannan	6.9 × 10^3^
BT3780	β-1,2-Mannobiose	2.4 × 10^3^
BT3780	β-1,2-Mannotriose	3.5 × 10^3^
BT3780	β-1,2-Mannotetraose	5.1 × 10^3^
BT3780	Man-β1,4-Glc	No activity*^a^*
BT3780	Man-β1,4-GlcNAc	No activity
BT3780	Man-β1,4-GlcNAc-β1,4-GlcNAc	No activity
BT3780	β1,4-Man oligosaccharides DP 2–6	No activity
BT3780	GlcNAc-β1,4-GlcNAc	No activity
BT3780	Man-1-phosphate	No activity
BACOVA_03624	*Candida* mannan	6.1 × 10^3^
BACOVA_03624	Man-β1,4-Glc	No activity

*^a^* For substrates where the two enzymes displayed no activity, assays were carried out in both 20 mm Na-Hepes and 50 mm sodium phosphate buffers, pH 7.5.

To explore further the specificity of BT3780, the catalytic activity of the enzyme against β1,2-mannooligosaccharides was determined. The data in Table 2 show that the enzyme exhibited low activity against the oligosaccharides with a *K_m_* that was too high to quantify, indicating weak affinity for these substrates. Given that the BT3780 displayed similar *k*_cat_/*K_m_* values for oligosaccharides with a degree of polymerization ranging from 2 to 4, the enzyme appears to contain only two subsites: −1 and +1. Phosphate did not influence the hydrolytic activity of BT3780, and mannose-1-phosphate did not participate in reverse phosphorolysis reactions with a range of sugars to generate β-linked disaccharides (data not shown). GH130 mannose-phosphorylases invert the anomeric configuration of the phosphorylated mannose residue generating α-mannose-1-phosphate (16). NMR analysis of the reaction products generated by BT3780 was used to determine whether the enzyme also hydrolyzed mannosidic linkages through a single displacement (inverting) mechanism. The data in Fig. 3 show that the initial product was α-d-mannose, which subsequently mutarotated to a 2:1 ratio of the α- and β-anomers of the sugar. *C. albicans* mannan polysaccharide was used as the substrate for the NMR experiment, which contributes a number of other features to the NMR spectra, including a peak at 4.99 ppm. The increase of this peak with incubation time likely corresponds to α-mannose side chains that are not capped by β-1,2 mannosides, which accumulate as digestion with BT3780 proceeds (30). These data show that both the glycoside phosphorylases and the glycoside hydrolases in family GH130 mediate bond cleavage though a single displacement inverting mechanism.

##### Crystal Structure of BT3780

**FIGURE 4.**
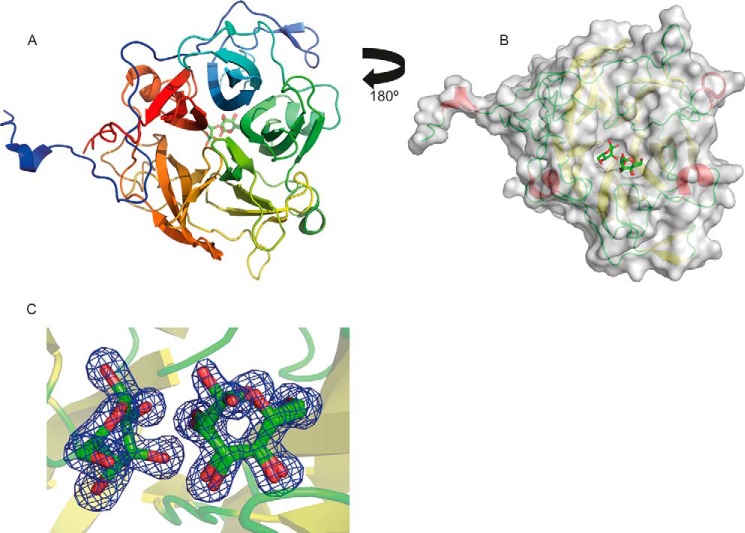
**Structure of BT3780.** *A* is a schematic of BT3780 revealing the five-bladed propellerfold ramp colored *blue* at the N terminus to *red* at the C terminus. The ligand is shown in background as stick representation with *green* carbon atoms, *red* oxygen atoms, and *blue* nitrogen atoms. *B* shows a surface representation of BT3780 with mannose residues bound in the active site pocket comprising the +1 and −1 subsites. The view is rotated 180° along a horizontal axis compared with *A* (ligand in foreground). The surface is represented semitransparent in *gray*. The secondary structure elements are represented in *red* for α-helices, *yellow* for β-strands, and *green* for loops. *C* shows the electron density map (2*F_o_* − *F_c_*) of the two mannose residues at 1.5 σ. The electron density is shown in *blue*.

To explore the structural basis for the catalytic activity and specificity of BT3780, the crystal structure of the enzyme was determined. Residues 17–384 (the first 16 residues is a cleaved signal peptide, and residue 383 is the C-terminal Pro) were built into the 1.35 Å electron density map (Table 1 and Fig. 4*A*). The β-mannosidase displayed a five-bladed β-propeller fold. Each blade consists of a β-sheet generally comprising four antiparallel strands, although blade 5 only contained three β-strands. Blade 4 contains a loop insertion in β-strand 1 extending from Cys^269^ to Ala^273^. The blades are arranged radially around the central axis and are strongly twisted. The β-sheets from the five blades pack face to face, with hydrophobic interactions and hydrogen bonds as observed for other β-propeller proteins. The propeller has a cylindrical shape with diameter and height of ∼40 Å and is present as a monomer in solution (data not shown). Most β-propeller proteins are “closed” by the completion of the C-terminal four-stranded sheet through incorporation of a strand from the N terminus, or vice versa, colloquially termed “molecular Velcro.” This is believed to provide considerable stabilization to the fold (31). Non-Velcroed propellers are rare, having primarily been described only for the seven-bladed prolyloligopeptidase, where the resultant flexibility is believed to facilitate substrate transfer (32). In BT3780 blades 1 and 5 are derived exclusively from N- and C-terminal sequences, respectively, and thus, no classical “Velcro” is present. There are, however, three hydrogen bonds between the N- and C-terminal blades. Of more significance is the long N-terminal loop extending from residues 17 to 70, which makes numerous polar contacts with the C-terminal blade and thus confers considerable stabilization of the protein fold.

BT3780 exhibits structural similarity to a range of proteins that display a five-bladed β-propeller fold, including several members of GH130. The closest homolog used as molecular replacement model is with the *B. ovatus* enzyme BACOVA_03624 (PDB code 3QC2, *z* score of 66, and root mean square deviation of 0.4 Å over 357 Cα atoms with sequence identity of 86%), which was previously shown to crystalize as a monomer. Both proteins are very similar, with identical arrangement of β-strands in the five blades. Inspection of the secondary structure of the only cytoplasmic GH130 *Bacteroides fragilis* β-1,4-mannosylglucose phosphorylase BF0772 (PDB code 3WAS) reveals an extended α-helix at the N and C termini. These helices mediate protomer interactions in the *B. fragilis* enzyme leading to a hexameric structure (20). The absence of these helices in BT3780 and BACOVA_03624 explains why these β-1,2-mannosidases are monomers, although in the GH130 Man-β1,4-GlcNAc phosphorylase UhgbMP, the hexameric structure is mediated by interactions between the β-propellers (19).

##### Active Site of BT3780

**FIGURE 5.**
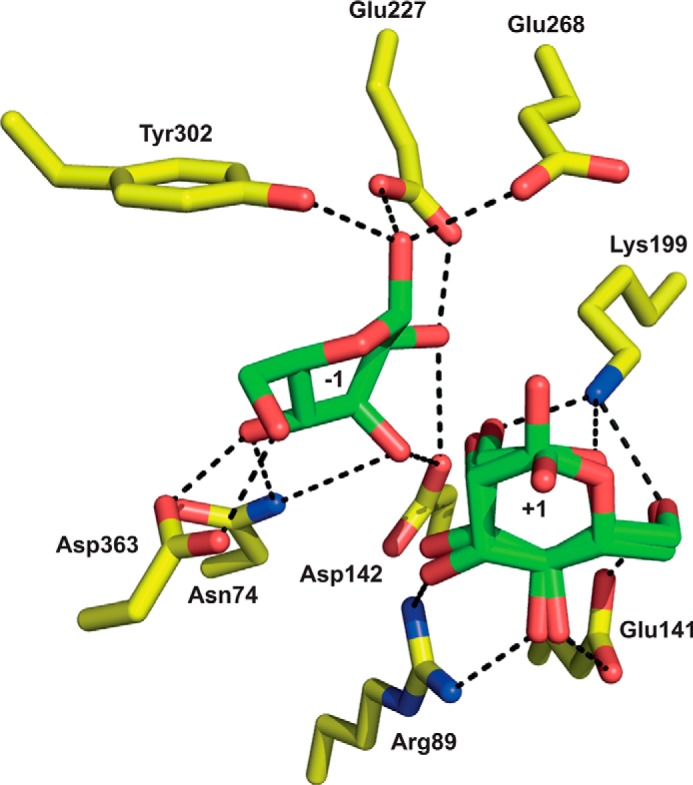
**The active site of BT3780.** The figure shows the three-dimensional position of the amino acids (carbons colored *yellow*) in the structure of BT3780 that make polar interactions (indicated by *black dashed lines*) with mannose (carbons colored *slate green*) bound in the −1 and +1 subsites.

An extended pocket is located in the center of the β-propeller with all five blades contributing to the topology of the pocket. The pocket houses two mannose residues (the crystal structure of BT3780 was obtained in the presence of mannose), and its location corresponds to the active site of BF0772 and thus fulfills the same function in the *B. thetaiotaomicron* enzyme (Fig. 4*B*). The mannose residue in the +1 subsite (Man2) adopts a classic ^4^*C*_1_ chair conformation. In the −1 subsite (active site) (33); however, the mannose (Man1) is in a *B*_2,5_/^O^*S*_2_ conformation, typical of the geometry adopted by the oxocarbenium ion transition state in β-mannanases and β-mannosidases (Fig. 4*C*) (34, 35). The interactions between the enzyme and products bound at the −1 and +1 subsites are detailed in Fig. 5. O_1_ of Man1 makes polar contacts with the side chains of Glu^227^, Glu^268^, and Tyr^302^; O_2_ interacts with Asp^142^ and Glu^227^; O_3_ forms a hydrogen bond with Asp^142^ and Asn^74^; O_4_ also makes a polar contact with Asn^74^, and Asp^363^ forms bidentate interactions with O_4_ and O_6_. At the +1 subsite, Man2 recognition is dominated by Lys^199^, which interacts with O_2_, O_6_, and the endocyclic oxygen; Arg^89^ makes three hydrogen bonds with O_3_ and O_4_, whereas Glu^141^ interacts with O_4_ and O_6_. At the −1 subsite, a cradle of aromatic residues, Tyr^302^, Tyr^338^, and Phe^344^, makes hydrophobic interactions with Man1, whereas at the +1 subsite, Trp^160^ and a few aliphatic residues make apolar contacts with Man2. BT3780 does not make interactions with O_1_ of Man2, and thus both the α and β anomers are evident. O_1_ of both anomers of Man2 are pointing directly into solvent, explaining why BT3780 can attack the terminal β-1,2-Man linkages in highly complex polysaccharides such as mannan in the cell wall of *C. albicans*. Indeed, the solvent exposure of both anomers of Man2 indicates that BT3780 can hydrolyze linkages where the +1 sugar is linked α or β to the mannan side chains. This is significant because BT3780 can fully hydrolyze the β-1,2-Man structures that cap the mannan side chains and thus expose the α-linked mannosidic linkages to attack by α-mannosidases such as BT3774 (see above). The importance of these active site residues in the activity of BT3780 was explored by site-directed mutagenesis. The data in Table 3 show that Asp^142^, Lys^199^, and Asp^363^ all played a critical role in catalysis because substitution of these residues led to complete loss in mannosidase activity. The 40-fold reduction in *k*_cat_/*K_m_* of N74A against *Candida* mannan, compared with the wild type enzyme, indicates that Asn^74^ also contributes to the activity of BT3780. R89A, however, was only 7-fold less active than wild type BT3780, and thus, although Arg^89^ is highly conserved (see below) and apparently makes several interactions with substrate at the +1 subsite, this residue makes little contribution to the catalytic efficiency of the enzyme.

**TABLE 3:** **Relative catalytic activity of mutants of BT3780** The activity of the enzymes were determined using 1 mg/ml *C. albicans* mannan as the substrate.

Variant of BT3780	Relative activity
Wild type	1.0
N74A	0.15
R89A	0.025
E141Q	No activity
D142N	No activity
K199A	No activity
E227Q	0.032
E268A	0.005
E268Q	0.004
E227Q/E268Q	No activity
Y302A	0.06
F344A	0.31
D363A	No activity

##### The Catalytic Apparatus of BT3780

A distinctive feature of the active site of BT3780 is the lack of residues that could easily fulfill the role of a classical catalytic general acid. Asp^142^ is the only potential catalytic acid because it is invariant in GH130 (see below) and is closest to the scissile bond. The residue, however, is too distant (4.8 Å) to make direct polar contact with the glycosidic oxygen (Fig. 5). It is possible that this acidic residue donates a proton to the glycosidic oxygen via solvent. It has been proposed that the equivalent residue in the Man-β1,4-Glc and Man-β1,4-GlcNAc phosphorylases BF0772 and UhgbMP (Asp^131^ and Asp^104^, respectively) activates a proton relay culminating in the protonation of the glycosidic oxygen (19, 20). In this mechanism the glycosidic oxygen abstracts the proton from O_3_, which in turn receives a proton from Oδ2 of the aspartate. Support for this proposal is provided by BF0772 and UhgbMP, in which O_3_ of Man1 is within hydrogen bonding distance to O_4_ of the Glc and GlcNAc, respectively, which occupy the +1 subsite. The importance of the carboxylate of the aspartate is demonstrated by the catalytically inactive mutant D142N. It should be emphasized that to date only a direct interaction between the catalytic acid and the glycosidic oxygen has been observed. It should be noted, however, that in other glycoside hydrolases the substrate can provide direct catalytic nucleophilic assistance, typically the carbonyl of the C2 acetamido group in GH18 and GH20 *N*-acetylglucosaminidases (36) but also the O_2_ of the active site mannose in GH99 endo-α1,2-mannosidases (37). Thus, the provision of catalytic groups by the substrate is not without precedent. The environment of Asp^142^ in BT3780 is not obviously apolar, and there is no residue that could act as a direct p*K_a_* modulator of the aspartate. Thus, the identity of the catalytic acid of BT3780 remains opaque. It is possible that BT3780 lacks a canonical catalytic acid, which may explain the very modest activity displayed by the enzyme.

In GH130 mannoside phosphorylases, the phosphate acts as the nucleophile mounting a direct nucleophilic attack on the anomeric carbon. In inverting glycoside hydrolases, an activated water molecule acts as the catalytic nucleophile. In BT3780 the glutamates Glu^227^ and Glu^268^ lie on the appropriate face of Man1 to facilitate nucleophilic attack by water at the anomeric center. The carboxylates of both Glu^227^ and Glu^268^ hydrogen bond to the α-linked O_1_ hydroxyl of the mannoside with distances of ∼2.7 Å (Fig. 5), and either could potentially activate a water molecule in the single-displacement mechanism. A similar pair of carboxylic amino acids also hydrogen bond to the catalytic nucleophilic water of inverting GH67 α-glucuronidases, making the assignment of the catalytic base difficult (38). The mutation of both glutamates in BT3780 to Gln resulted in a relatively modest reduction in catalytic activity, 30-fold for Glu^227^ and 200-fold for Glu^268^. Substitution of the catalytic residues of glycoside hydrolases generally results in an effectively inactive enzyme (39, 40); however, mutation of the candidate catalytic base of several inverting glycanases did not result in complete loss of enzymatic activity enzymatic activity (41, 42). It is possible that the Glu^227^/Glu^268^ pair influences the position and pK_a_ of the other, and thus both residues may contribute to the function of the catalytic base. Mutation of either of these residues could increase the polarity of the other, enabling it to fulfill a catalytic base function. Indeed, in retaining glycanases mutating the catalytic nucleophile or occupation of the active site with substrate results in the deprotonation of the catalytic acid/base, which thus functions exclusively as a base (43, 44). The redundant identity of the catalytic base in BT3780 is consistent with the complete loss in activity only when both glutamates were substituted for glutamine (Table 3).

##### Functional Phylogeny

**FIGURE 6.**
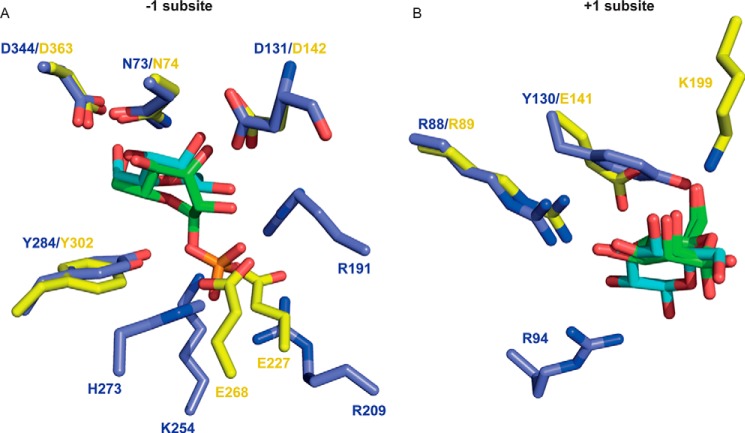
**Comparison of the active site of BT3780 and a β-mannoside phosphorylase.** The figure shows an overlay of the active site of BT3780 (*yellow* carbons) with the β-mannoside phosphorylase BF0772 (PDB code 3WAS, *blue* carbons). BT3780 is in complex with two mannose residues (*green*), whereas BF0772 is bound to Man-β1,4-Glc (*cyan* carbons) and phosphate (*orange*). *A* and *B* show the −1 and +1 subsites, respectively.

**FIGURE 7.**
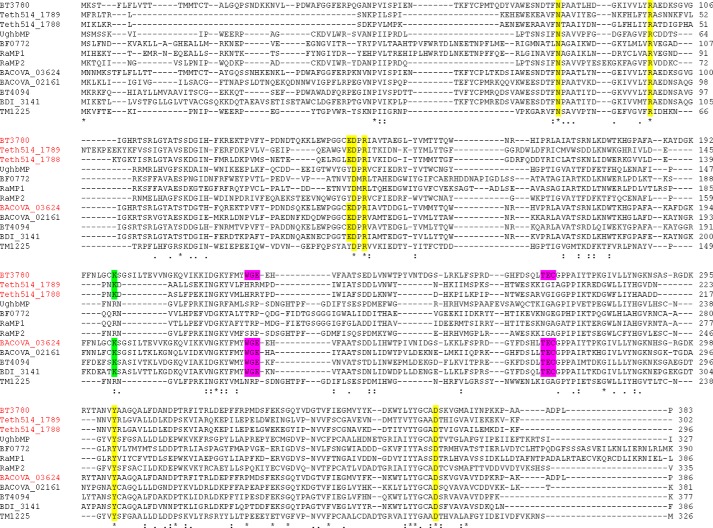
**Alignment of GH130 sequences.** Members of GH130 that have been characterized and/or whose three-dimensional structure has been determined are shown. Enzymes in *red* and *black* cleave β-1,2-Man and β-1,4-Man linkages, respectively. Residues that are conserved and contribute to substrate binding in BT3780 are highlighted in *yellow*, the motifs that contain the residues that are predicted to comprise the catalytic base of β-mannosidases are in *magenta*, and the lysine that confers specificity for β-1,2-Man linkages is in *green*.

This report provides functional insight into the phylogeny of GH130. In a previous study this family was classified into two distinct subfamilies, GH130_1 and GH130_2, whereas the other sequences were too heterogeneous to be grouped into a single subfamily and were thus defined as GH130_NC (18). To date, the enzymes characterized from GH130_1 and GH130_2 are mannoside phosphorylases. In these enzymes, phosphate, which comprises the catalytic nucleophile, is positioned below the α-face of the −1 Man and makes polar interactions with three basic residues (Fig. 6). Members of the GH130_NC grouping, which includes BT3780 and BACOVA_03624, lacked the three basic residues that interact with phosphate in mannoside phosphorylases, and it was proposed that these enzymes might be glycoside hydrolases (18). The data published here are entirely consistent withthis hypothesis. To evaluate further this proposal, the biochemical properties of another member of GH130_NC, BACOVA_03624 from *B. ovatus*, were evaluated. The data show that the enzyme is also a β-1,2-mannosidase (Table 2), supporting the functional classification of the GH130_NC grouping as β-mannosidases. Apart from the acidic/basic residue substitution, the other residues that bind to the −1 Man in BT3780 are conserved in mannoside phosphorylases (Figs. 6*A* and 7). This suggests that the boat conformation of the bound mannose within GH130 enzymes is independent of hydrolytic or phosphorolytic cleavage of the glycosidic bond.

Another distinguishing feature between GH130_NC and the two GH130 subfamilies GH130_1 and GH130_2, is a tyrosine/glutamate substitution adjacent to the putative catalytic acid. It was previously proposed that the glutamate was the catalytic base (18), when no GH130 structure in complex with substrates was available. The residue in BT3780 (Glu^141^), however, interacts with O_4_ and O_6_ of mannose at the +1 subsite, and these hydrogen bonds replace the partial hydrophobic platform afforded by the tyrosine in GH130_1/2 enzymes (Fig. 6*B*). This glutamate is thus not in an appropriate position to act as the catalytic base.

In general, the +1 subsite of GH130 enzymes are not highly conserved. This likely reflects the different specificities displayed by this family in which the scissile linkage can be β-1,2 or β-1,4 and the +1 sugar Glc, Man, or GlcNAc. This is exemplified by BT3780 and BF0772, which target β-1,2-Man-Man and β-1,4-Man-Glc linkages, respectively. Consequently, the sugars in the +1 subsite of these two enzymes are in a perpendicular orientation and rotated with respect to each other. Nevertheless Arg^89^, which interacts with O_3_ and O_4_ of Man2 in BT3780, is conserved in BF0772 where the basic residue makes hydrogen bonds with equatorial O_2_ and O_3_ of Glc (Fig. 6*B*) and explains why the *B. fragilis* enzyme does not hydrolyze Man-β-1,4-Man linkages. The other residue that makes polar contacts with Glc at the +1 subsite of BF0772 is Arg^94^. Although this amino acid is conserved in BT3780 (Arg^101^), the basic residue does not interact with Man2 and is not invariant in GH130 (Fig. 7).

A key specificity determinant in BT3780 is likely to be Lys^199^. The lysine is the only residue that interacts with O_2_ of Man2 (Fig. 5) and thus is likely to confer specificity for Man-β1,2-Man linkages. Indeed, Lys^199^ is conserved in the other three enzymes that are known to target Man-β1,2-Man linkages (Fig. 7). These enzymes display mannosidase (BACOVA_03624) (Table 2) and mannoside phosphorylase (Teth514_1788 and Teth514_1789) activities (13). The basic residue, however, is not conserved in mannoside phophorylases that target β1,4-Man linkages, irrespective of the nature of the +1 sugar (Fig. 7).

## Discussion

This report supports the hypothesis that the GH130 family contains glycoside hydrolases in addition to mannoside phosphorylases. The two characterized GH130 mannosidases target Man-β1,2-Man linkages. The predicted GH130 glycoside hydrolases, based on substitution of basic residues with glutamates, also contain the lysine β1,2-Man specificity determinant. It remains unclear whether other GH130 glycoside hydrolases display additional specificities. The catalytic efficiency of BT3780 is modest, ∼1000-fold lower than typical glycosidases. It is possible that this reflects in part the absence of a canonical catalytic acid leading to a low *k*_cat_. In a recent study, it was shown that *B. thetaiotaomicron* degrades yeast mannan through a selfish mechanism, which requires slow acting surface enzymes exemplified by the β1,2-mannosidase described here (21). It is also possible that although BT3780 is active on the Man-β1,2-Man oligosaccharides of *C. albicans* mannan, its true substrate is a different fungal cell wall, which contains β-Man in an alternative context. Indeed, it is also possible that other enzymes contribute to the degradation of *C. albicans* mannan. Although few data are available on the fine structure of the cell wall mannans of other gut fungi, it should be noted that the *Agaricus brasiliensis* cell wall contains sulfated β1,3-Glc-β1,2-Man (45), whereas *Hericium erinaceus* produces β-1,3-branched-β-1,2-mannan (46). Although the biological rationale for both β-mannosidases and β-mannoside phosphorylases in GH130 is unclear, it should be noted that the phosphorylases are cytoplasmic, whereas the hydrolases are secreted. These different cellular locations suggest that the mannosidases target complex substrates that cannot be imported into the periplasm, whereas the phosphorylases, using intracellular inorganic phosphate, cleave and activate the glycone sugar enabling the phosphorylated molecule to enter cytoplasmic metabolic pathways.

Based on the presence of signal peptide, the absence of the three basic residues that interact with phosphate in mannoside phosphorylases, conservation of the motifs comprising the predicted catalytic base of β-mannosidases, and of the lysine that confers linkage specificity, it is thus now possible to predict the catalytic mechanism and the β-mannoside substrates of the hundreds of uncharacterized enzymes within GH130. Because many of these enzymes, exemplified by BT3780 and BACOVA_03624, are highly prevalent in the human gut microbiome, these data provide insight into glycan utilization in this microbial ecosystem.

## Author Contributions

F. C. determined the biochemistry of BT3780. A. B. solved the crystal structure of BT3780. S. L. expressed and purified BACOVA_03624. A. M. D. prepared substrates for the work. G. J. D. provided intellectual insight into enzyme function. H. J. G. supervised the work and contributed to writing the manuscript. G. P.-V. contributed to writing the paper and to characterizing BACOVA_03624. E. C. L. crystallized BT3780, contributed to the design of experiments, and contributed to the writing of the paper. All authors reviewed the results and approved the final version of the manuscript.

## Footnotes

3 The abbreviations used are:

GH: glycoside hydrolase
PUL: polysaccharide utilization locus
PDB: Protein Data Bank
HPAEC: high pressure anion exchange chromatography.
